# New Breakthroughs and Innovation Modes in English Education in Post-pandemic Era

**DOI:** 10.3389/fpsyg.2022.839440

**Published:** 2022-02-11

**Authors:** Yumin Shen, Hongyu Guo

**Affiliations:** ^1^School of Foreign Languages, Zhejiang Gongshang University, Hangzhou, China; ^2^Graduate School of Education, University of Perpetual Help System DALTA, Metro Manila, Philippines

**Keywords:** post-pandemic, english teaching, dual-modality, facial expression, physiological signal

## Abstract

The outbreak of COVID-19 has brought drastic changes to English teaching as it has shifted from the offline mode before the pandemic to the online mode during the pandemic. However, in the post-pandemic era, there are still many problems in the effective implementation of the process of English teaching, leading to the inability of achieving better results in the quality and efficiency of English teaching and effective cultivation of students’ practical application ability. In recent years, English speaking has attracted the attention of experts and scholars. Therefore, this study constructs an interactive English-speaking practice scene based on a virtual character. A dual-modality emotion recognition method is proposed that mainly recognizes and analyzes facial expressions and physiological signals of students and the virtual character in each scene. Thereafter, the system adjusts the difficulty of the conversation according to the current state of students, toward making the conversation more conducive to the students’ understanding and gradually improving their English-speaking ability. The simulation compares nine facial expressions based on the eNTERFACE05 and CAS-PEAL datasets, which shows that the emotion recognition method proposed in this manuscript can effectively recognize students’ emotions in interactive English-speaking practice and reduce the recognition time to a great extent. The recognition accuracy of the nine facial expressions was close to 90% for the dual-modality emotion recognition method in the eNTERFACE05 dataset, and the recognition accuracy of the dual-modality emotion recognition method was significantly improved with an average improvement of approximately 5%.

## Introduction

At the beginning of 2020, COVID-19 hit the world and has since impacted all aspects of society, including a change in students’ thinking and cognition (Baloch et al., 2020). In the post-pandemic era, great significance must be attached to the crucial role of English in students’ future development, working toward changing the teaching mode, adopting scientific and effective English teaching ideas, and seeking new breakthroughs and innovative models of English teaching, to continuously improve students’ English skills and literacy (Tarpey, 2009; Kelly et al., 2013).

Currently in China, learning English at the basic level is still primarily based on the study of the written language, where students need to master formal English language logic and vocabulary. Both textbook requirements and entrance examinations are based on this standard, with little attention being paid to the content of spoken English (Chen and Yu, 2013; Liao and Li, 2020). Many students mistakenly believe that the English they have learned are the common language of the native English-speaking countries. Therefore, in teaching spoken English, many students show a careless attitude as they believe that their level of English is completely capable of communicating with native English speakers (Du and Liang, 2021). In the process of teaching spoken English, with the lack of certain strategies and exploration of new models with teachers, students’ interest in learning cannot be improved effectively. Particularly in the post-pandemic era, more attention needs to be paid to the practice of spoken English (Cheng and Zhang, 2021).

Any language needs the support of expressions, and English is no exception. Facial expressions, voice intonation, and body posture can convey different emotions. Therefore, recognizing different people’s emotions has become a key factor affecting the development of human–computer interaction and intelligence (Samara et al., 2019; Hu et al., 2021; Nayak et al., 2021). Generally, emotional information contained in the face is the most direct and abundant. Many studies have shown that despite differences in skin color, language, and social status, the basic patterns of facial expression and facial muscle movement in humans are generally consistent (de la Rosa et al., 2018). Relevant research shows that actual language accounts for only 35%, whereas non-verbal signals account for 65% of the impact of human expressions (Feng et al., 2019). Therefore, facial expressions are an important link for students to express their emotions. Virtual characters are introduced to help improve students’ learning efficacy (Mousas et al., 2018). In teaching practice, the role of virtual characters usually includes tutors, companions, and guides. In order to improve the emotional interaction of the virtual character, the facial expression data during the conversation between students and the virtual character is collected by a camera. The practicability of spoken English teaching can be enhanced by recognizing facial emotions and providing timely feedback.

Unlike during the COVID-19, students can attend classes in the post-pandemic era, which provides new opportunities for speaking. The emergence of artificial intelligence and other technologies provides technical support for creating new intelligent teaching environments and promoting the reform and breakthrough of English teaching methods (Bin and Mandal, 2019; Zheng and Shi, 2020). The most important thing in speaking English is to be able to speak out boldly. Conversation with native English speakers is an effective way to improve their English-speaking abilities. Given the financial limitations of different schools, it is impossible for every school to have foreign teachers to teach English. The interactive English-speaking practice system can talk with students through virtual characters, recognize students’ facial expressions in the conversation, judge whether students have mastered the conversation content, understand the intention of students from their expressions, and thereafter intelligently guide the conversations for students. For example, when students frown to show that they do not understand the content of the conversation, the system can gradually guide the conversation with similar meanings in an alternate way.

The main contributions of this study are summarized as follows: (i) an interactive English-speaking practice scene based on a virtual character is constructed to improve the ability of students to speak English; and (ii) a dual-modality emotion recognition method is proposed.

The remainder of this manuscript is organized as follows. Section “Related Work” reviews the related work. In section “Dual-Modality Emotion Recognition,” the dual-modality emotion recognition method is presented. The simulation results are presented in section “Simulation and Results Analysis.” Finally, section “Discussion” concludes the manuscript.

## Related Work

The continuous innovation and development in information technology have also impacted English education. The traditional uniform English education mode cannot consider the shortcomings of individual differences among students. The mode of English education needs to break through and innovate, and use information technology to stimulate students’ interest in learning so that English education can enter a new stage. In a recent study, the authors analyzed the higher education informatization-based English innovation teaching mode (Zhu, 2018). In order to quantitatively evaluate and analyze the effect of college English teaching innovation reform, a curriculum thinking-based evaluation model of college English teaching innovation reform was proposed to establish a large data analysis model of the effect of college English teaching innovation reform (Zhang and Zhan, 2020). In an English teaching course, the application of the whole-brain theory in English teaching was explored through a questionnaire survey of teachers and students in English classes (Mao and Zhang, 2018). In English translation, the author discussed the theoretical and methodological significance of translation methods in the study of multilingual user language innovation and the study of world English (Wei, 2020). With the development of information technology, the authors explored the impact of information technology resources on the innovation performance of higher education (Maulani et al., 2021). Considering 5G, artificial intelligence, and education, the author introduced 5G technology into English-speaking teaching, explored a new English-speaking teaching model through case design, summarized its advantages, and presented solutions to its shortcomings (Sun, 2021). To explore the characteristics of English teaching, the author established an innovative English teaching management model and investigated English teaching from the perspective of innovative management (Sun, 2017). Combining game theory and English education, the author established a game model and an evolutionary game model to analyze the necessity of the development of Chinese English cross-cultural awareness, and to make the best strategy choices (Li, 2016). In English teaching reform, the current situation of English teaching was discussed, and the development and reform of English teaching paradigms were analyzed to promote the professional development of English teaching (Zhao, 2017). Combining English theory and practice, the optimal application and innovation model of network resources in a college English-hearing class was analyzed (Yan et al., 2017).

Considering some specific scenes of human–computer interaction, such as classroom listening, fatigue driving detection, and other scenes where only facial expressions can be obtained, it is necessary to use facial features for emotion recognition. In hierarchical group-level emotion recognition, a novel method for group-level emotion recognition was proposed (Fujii et al., 2021). In emotion recognition of facial images, a convolutional neural network (CNN)-based deep learning technique was proposed to classify emotions from facial images (Khattak et al., 2021). A hybrid classifier could be used for emotion recognition, a novel classifier-based text-independent and speaker-independent emotion recognition system was proposed (Shahin et al., 2019). In neural networks, a single deep CNN-based model was proposed to recognize facial expressions (Jain et al., 2019). In automatic recognition of emotions, the authors used migratory learning to generate models of specific subjects to extract emotional content from facial images in the valence/wake dimension (Rescigno et al., 2020). In dual-channel emoticon recognition, machine learning theory and a philosophical-thought-based feature fusion dual-channel emoticon recognition algorithm were proposed (Song, 2021). In the facial emotion recognition system, the authors used feature extraction based on scale-invariant feature transformation to extract features from face points (Sreedharan et al., 2018). With the enhancements on the Internet of medical things, the authors introduced an Internet of Medical Things-based face emotion detection and recognition system (Rathour et al., 2021). In human–robot interaction system, the authors proposed a multimodal emotional recognition method to build a system with a low sense of disharmony (Tan et al., 2021). At present, facial expression recognition is seldom applied to English teaching; hence, this study carries out research on it.

## Dual-Modality Emotion Recognition

Although facial expressions can directly reflect emotional changes, visual perception alone cannot perceive all emotional changes. Therefore, some scholars have proposed analyzing the potential emotional states of the human body through physiological signals to compensate for the deficiency of facial expressions in single-modal emotion recognition.

**FIGURE 1:**
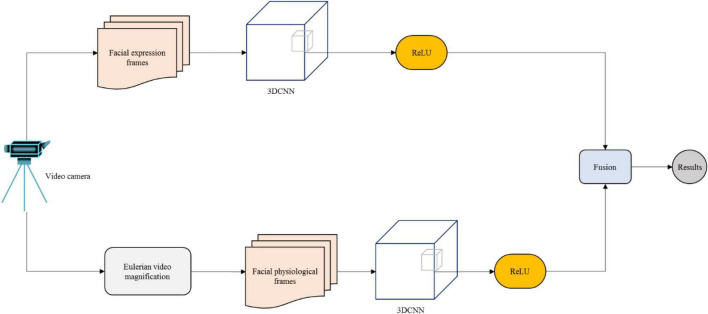
Dual-modality emotion recognition framework.

The proposed dual-modality method combines facial expression features and hidden physiological features of the face in real-time video for emotion recognition. The students’ expressions are obtained through the camera; thereafter, face detection and clipping are carried out on the facial videos. Features of facial appearance and facial physiology are extracted, and the final emotion classification is carried out by combining the classification results of the dual-modality. The model framework is illustrated in Figure 1.

### Video Pre-processing

Since the size of the collected original video is too large and contains a lot of unnecessary background information, such as tables and chairs, it will have a certain impact on the training efficiency and accuracy of the dual-modality emotion recognition model. Therefore, first, face detection and clipping should be carried out on the video, and the video should be divided into frames after clipping. In addition, to increase the diversity of data and improve the generalizability of the model, the facial images extracted from the video are processed by data enhancement, including translation and flipping.

Owing to the periodic contraction and expansion of the heart, the blood volume of the face changes accordingly, resulting in subtle color changes in the face depending on how the blood volume of the face and other tissues absorb light differently. To obtain the physiological signals hidden in the videos of students’ faces in interactive English-speaking practice, Eulerian video magnification (EVM) (Wadhwa et al., 2017) is used to magnify the color of facial videos after the facial image is clipped. This is done to enhance the slight color change of the face and facilitate the extraction of facial physiological features.

Eulerian video magnification first decomposes the input video images into video images of different scales, which is equivalent to the spatial filtering of videos. Then, the spatially filtered video image is processed by time-domain band-pass filtering and multiplied by a magnification factor to obtain the magnified video image. Finally, the video image obtained in the first two steps is reconstructed to obtain an enlarged video image.

### Feature Extraction of Students’ Emotion

The extraction methods for the facial expressions of students are classified, which can be roughly divided into three types: overall feature extraction, local feature extraction, and geometric feature extraction. As the name implies, the method of overall feature extraction is to take the students’ facial expressions as a whole for scientific research and analysis in the research on students’ facial feature extraction. The purpose of this study is to effectively extract the overall differences between various expressions, such as anger, happiness, and excitement. In contrast, the local analysis method is the first to separate the facial features of the students, making a differential analysis based on the significance of each detail of the facial features of the students in expression analysis.

### Three-Dimensional Convolutional Neural Networks-Based Students’ Facial Emotion Recognition

In this study, the recognition of students’ facial emotion is performed based on CNNs that have the ability of representation learning and can carry out shift-invariant classification according to each hidden layer when inputting data. The CNN designed in this study contains four convolution layers, each of which is composed of several smaller convolution layers, thus reducing the amount of computation.

Taking rectified linear unit (ReLU) as the activation function, it can perform a nonlinear mapping on the result of the product of the weight and input value. The advantage of this is that it can make CNN have the ability of nonlinear modeling after adding the activation function. Thus, we can convert the result obtained by operation into a value in an input field. ReLU was introduced as an activation function for the following reasons: (i) if sigmoid and other functions are used, the amount of calculation is large when calculating the activation function. When calculating the error gradient by back propagation, the derivation involves division, which requires a considerable amount of calculation. However, using the ReLU activation function saves extensive calculations in the entire process. (ii) When the sigmoid function propagates back, the gradient disappears easily; thus, the training of the deep-layered network cannot be completed. (iii) ReLU will make the output of some neurons 0, thus resulting in the sparsity of the network, reducing the interdependence of parameters and alleviating the occurrence of overfitting problems.

In addition, the direct use of convolution features in classification can lead to disastrous consequences such as an exponential increase in the amount of computation and the consequent risk of overfitting. Therefore, we perform pooling for this problem, that is, before using a neural network to conduct a series of sample classifications each time, the pooling process must be processed for sample data features after neural network convolution. The pooling operation is the same as the convolution operation and can be understood as the same form of downsampling. It divides the input sample data into several areas. Thereafter, there are two processing methods for several areas: taking their maximum value or taking their average value. Pooling not only reduces the amount of computation significantly but also avoids the risk of overfitting.

To prevent overfitting, dropouts are used in the emotion recognition of students. The application of dropout in CNN is mainly to randomly select a certain proportion of neurons in the full connection layer to participate in the computation of the network during each propagation of the neural network (including forward propagation and backward propagation), thus reducing the amount of computation and eliminating certain risks of overfitting. The batch normalization method is used to normalize the parameters of each layer of the convolution, which makes the CNN faster and more stable (Li et al., 2018).

Given the above, convolution and pooling operations are only applied to the feature extraction of two-dimensional static images in space. Since three-dimensional CNN (3DCNN) can effectively extract video space-time information, 3DCNN has attracted much attention in the field of video behavior recognition. 3DCNN is mainly used to extract dynamic images from real-time videos of students’ English-speaking practice. It adds an extra time dimension, which can be convolved and pooled in space-time (Wang et al., 2021). The 2DCNN takes multiple images as multiple channel inputs and outputs one feature of the output image each time. Therefore, the time information of the input signal is lost after each convolution operation. 3DCNN superimposes multiple images into a cube as a channel input and outputs the characteristics of multiple images to retain the time information of the input signal to extract the time characteristics of the sequence.

### The Dual-Modality Emotion Recognition Method

The fusion of multimodal data can be realized using different fusion methods. The best accuracy can be achieved using the appropriate fusion methods. Feature fusion is a common method that can connect features into a high-dimensional eigenvector and then send it to the classifier. However, with the addition of a large amount of information to the combined features, the training efficiency and computing resources will be significantly affected. Decision fusion assigns fusion weights to classifiers trained with different features for fusion to obtain the final discrimination results that not only integrate the results of different modes but also reduce the training burden.

The key step in decision fusion is the allocation of the weights of different modes. The common fusion criteria include the maximum, mean, and product criteria, but these methods are based on simple mathematical calculations and do not consider the possibility of weight allocation other than the criterion. For the weight allocation of dual-modality fusion, this manuscript proposes an automatic weight optimization method to find the optimal weight allocation scheme of the two modalities from a sufficient number of weight combinations, which can be defined as follows:


(1) $$y=\arg\text{max}\,\left(\alpha \,P_{f\,a\,c}+\beta \,P_{p\,h\,y}\right)$$

where *y* represents the prediction category, α represents the weight of the facial feature recognition results, and β represents the weight of the physiological feature recognition. α + β = 1, *P*_*fac*_ represents the category probability of the facial feature prediction results, and *P*_*phy*_ represents the category probability of the physiological feature prediction results.

The steps for the automatic weight optimization strategy are summarized as follows.

Step 1: Initialize the weight α and extract the category prediction *P*_*fac*_ and *P*_*phy*_ of the two modalities, and the real label value *y*′.

Step 2: Start from α = 0.0, and increase by 0.001, the maximum is 1.0.

Step 3: Calculate the prediction category *y* recurrently, and save the weights simultaneously.

Step 4: Compare *y* with *y*′ recurrently and calculate the accuracy.

Step 5: Select the highest accuracy and obtain the weight corresponding to the highest accuracy, that is, the optimal weight.

**FIGURE 2:**
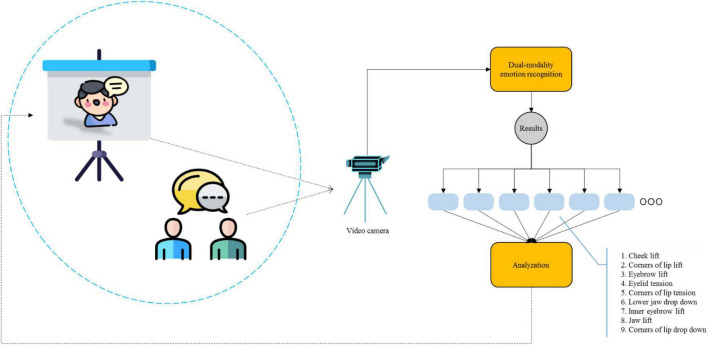
The overall framework of interactive English-speaking practice.

The overall framework of the system is shown in Figure 2. During the interactive English-speaking practice, students are arranged to have conversations with the virtual characters on the 3D projection screen in various scenarios to improve their English-speaking ability and achieve new breakthroughs in English education. The conversation between students and the virtual characters starts in a simple form. As the students gradually adapt to the pronunciations of the virtual character, the system adjusts the difficulty of the conversation through facial expressions and physiological signals of the students, and gradually guides the students to complete the conversation of each scene. For example, students may frown in a conversation as it may be difficult for them to understand the current conversation. The system will accordingly express what is to be said next in an alternate way that is easy for students to understand. Subsequently, the system will guide the content of the conversation as per the results of the students’ emotional recognition. Therefore, by applying the innovative mode of personalized conversation content through students’ emotion recognition, students can complete the conversation in each scene in an interactive manner, improving their English-speaking ability to a considerable extent.

## Simulation and Results Analysis

### Simulation Dataset

The simulation was driven on the eNTERFACE05 and CAS-PEAL datasets. The eNTERFACE05 dataset contains 44 subjects from 14 different nations. Each user expressed six emotions five times. When judged by professionals, the database contained 1,166 video sequences. Since Chinese students belong to the Asian population, a special dataset was selected to recognize the students, that is, the large-scale Chinese face dataset (CAS-PEAL).

### Parameters Setting

The simulation was performed on a 64-bit Ubuntu 20.04 operating system, using an NVIDIA GeForce RTX 3060 Ti graphics card for graphics processing unit (GPU) acceleration. The input size of the network was 3 32 224 224, where 3 represents three color channels, and 32 represents the number of frames input at one time. Based on the 3DCNN architecture, the network had eight convolution layers, five maximum pooling layers, and three fully connected layers. The number of convolution kernels of eight convolution layers was 64, 128, 128, 256, 256, 256, 512, and 512, respectively. The size of the convolution kernel was three, and the step size was one. The kernel size of the pool layer was two, and the step size was two. The number of output features of the first two full connection layers was 4,096, and the number of output features of the third full connection layer was the number of categories in the dataset. The pre-trained 3DCNN model was used for training. The loss function is a cross-entropy function. A random-gradient descent algorithm was used to optimize the loss function. A small batch training network with ten video clips was used. The initial learning rate was set at 0.0001. After every 10 epochs, the learning rate was reduced by 10 times, and a total of 50 epochs were trained.

### Comparison Analysis

To verify the performance of the proposed dual-modality emotion recognition method and its recognition accuracy and recognition time, group-level emotion recognition (GLER) (Fujii et al., 2021), deep learning using convolutional neural network (DL-CNN) (Khattak et al., 2021), and Gaussian mixture model and deep neural network (GMM-DNN) (Shahin et al., 2019) were compared on the eNTERFACE05 and CAS-PEAL datasets, respectively. The movement of facial muscles forms the facial expressions, which are divided into nine categories. In the interactive English-speaking practice scene, the cheek lift, corners of lip lift, and eyebrow lift indicate that the students have mastered the conversation with the virtual character, and the system will induce more difficult conversations for the students. Eyelid tension, corners of lip tension, and lower jaw drop down indicate that students have a basic understanding of the conversation with the virtual character, and the system will continue to guide the conversation with the same difficulty for students. The inner eyebrow lift, jaw lift, and corners of the lip drop-down indicate that the students do not understand the conversation with the virtual character, and the system will lower the difficulty and gradually guide the students to improve on the difficulty of the conversation.

**FIGURE 3:**
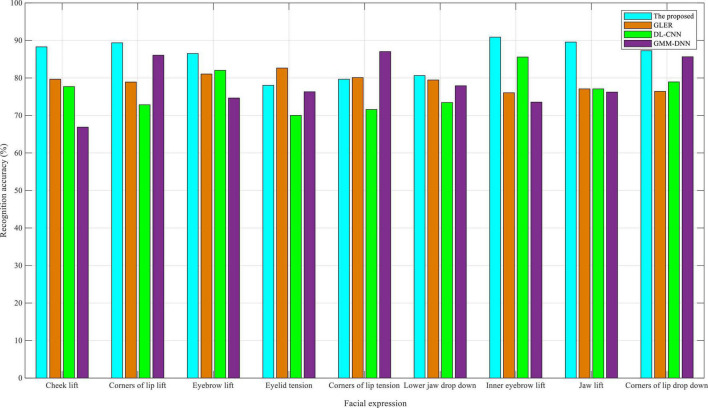
Comparison of recognition accuracy in eNTERFACE05 dataset.

As shown in Figure 3, in the eNTERFACE05 dataset, the recognition accuracy of nine facial expressions is close to 90% for the dual-modality emotion recognition method proposed in this manuscript. The reason why it does not exceed 90% is that there are fewer facial expressions of Asians in the eNTERFACE05 dataset, and the facial structure is more or less different, which is mainly because the eNTERFACE05 dataset is an emotional dataset, 42 subjects come from 14 different countries, of which 81% are men and the rest are women, 31% wear glasses, and 17% have beards. Despite this, the algorithm proposed in this study still achieves a good recognition accuracy. This also proves that even if the proposed method is applied to interactive English-speaking scenes in other countries, improved recognition accuracy can be achieved. By reviewing the other three baselines, GLER performed well in the recognition accuracy of facial expressions. DL-CNN had a high recognition accuracy in eyebrow-related expressions, whereas GMM-DNN had a good recognition accuracy in the corners of the lip; the recognition accuracy of some expressions even exceeds that of the proposed algorithm in this study.

**FIGURE 4:**
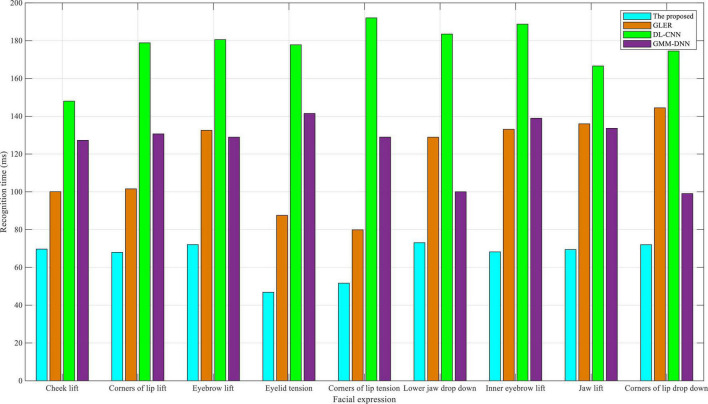
Comparison of recognition time in eNTERFACE05 dataset.

As shown in Figure 4, comparing the recognition time of nine facial expressions on the eNTERFACE05 dataset, the dual-modality emotion recognition method proposed in this manuscript always has the lowest recognition time of nine facial expressions, especially lift-related facial expressions. Time recognition plays an important role in English-speaking interactive practices. The length of the recognition time determines whether the system can timely guide appropriate conversations for students in the interaction. If the recognition time is too long, apart from causing the embarrassment of the interactive scene, the students may not be confident in their English-speaking ability. For the other three baselines, GLER had a lower recognition time in tension-related expressions, DL-CNN had a more balanced recognition time for all facial expressions, and GMM-DNN had a better recognition time in drop down-related expressions. In conclusion, the algorithm proposed in this study performs well in terms of recognition accuracy and recognition time on the eNTERFACE05 dataset, thus providing high recognition accuracy and low recognition time for English-speaking interactive practice.

**FIGURE 5:**
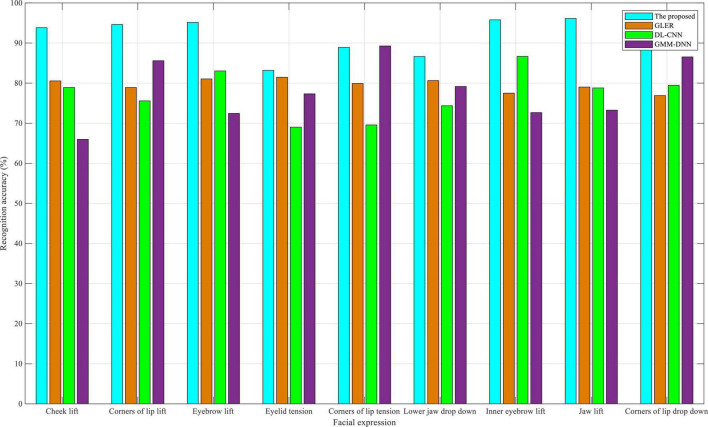
Comparison of recognition accuracy in CAS-PEAL dataset.

As indicated in Figure 5, in the CAS-PEAL dataset, the recognition accuracy of the algorithm proposed in this study is significantly improved with an average improvement of approximately 5%. This is mainly because the CAS-PEAL dataset is primarily an Asian face dataset, which has a high fit with the faces of students in the English-speaking interactive practice in this study; hence, the recognition accuracy is much higher than that of the eNTERFACE05 dataset. The improvement of recognition accuracy is of great help to students’ English-speaking interactive practice, as the high-precision recognition accuracy provides a more accurate guarantee for the system to guide the next conversation for students. Owing to the high recognition accuracy, the system can accurately capture and recognize the slightest change in students’ English-speaking interactive practice, which provides a favorable foundation for dual-modality emotion recognition to a great extent. The tension-related recognition accuracy of the GLER and DL-CNN algorithms was reduced along with the lift-related recognition accuracy of the GMM-DNN algorithm; however, the recognition of other facial expressions improved accordingly.

**FIGURE 6:**
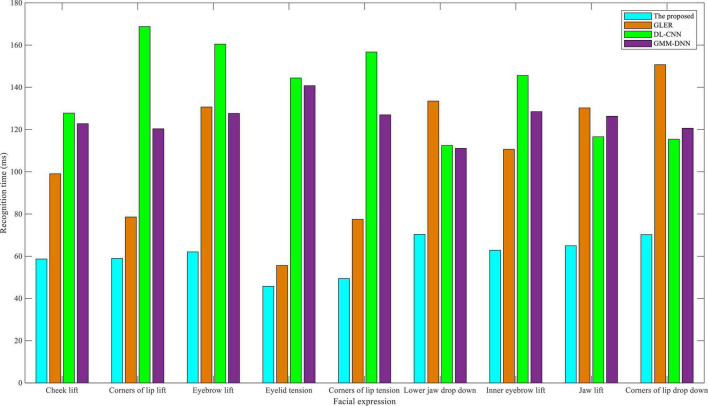
Comparison of recognition time in CAS-PEAL dataset.

Figure 6 indicates that in the CAS-PEAL dataset, the recognition time of the algorithm proposed in this study is always the shortest. GLER and GMM-DNN exhibit an increase in the drop down-related recognition time. In contrast, the recognition time of the DL-CNN algorithm was much lower than that of the eNTERFACE05 dataset. A short recognition time ensures the efficiency of the system, that is, students will have a small probability of lag in the system in interactive English-speaking practice. The interactive English-speaking practice system can analyze the facial expression of students in a timely manner and guide the conversation suitable for the current context for students, rather than making it difficult for students to understand the content of the conversation directly.

In summary, the algorithm proposed in this manuscript performs well in terms of emotion recognition accuracy and recognition time for the eNTERFACE05 and CAS-PEAL datasets. The two metrics promote the innovative model of English education and make a breakthrough in the multi-scene English-speaking interactive practice constructed in this study.

## Discussion

Information technology has promoted diversity and creative reform in English teaching, providing more possibilities for the innovation of English teaching models in the post-pandemic era. The proposed dual-modality method combines facial expression features and hidden facial physiological features in real-time videos for emotion recognition. After obtaining the physiological signals, EVM was used to magnify the color of the facial videos. During 3DCNN-based students’ facial emotion recognition, the ReLU activation function can perform a nonlinear mapping of the result of the product of weight and input value. 3DCNN was mainly used to extract dynamic images from real-time videos of students’ interactive English-speaking practice. The simulation was driven on the eNTERFACE05 and CAS-PEAL datasets, and its results demonstrate that the proposed emotion recognition method outperforms benchmarks in terms of recognition accuracy and recognition time. Thus, in this innovative model students’ English-speaking ability progressively improved through interactive practice, and they gradually adapted to the English language environment. This study only examined the recognition of facial expressions. In interactive English-speaking practice, the intonation of students’ English pronunciation is as important in reflecting students’ understanding of the conversation. At the same time, for students’ pronunciation, the system can simultaneously be programmed to point out and correct incorrect pronunciations in subsequent conversations.

## Data Availability Statement

The raw data supporting the conclusions of this article will be made available by the authors, without undue reservation.

## Author Contributions

YS contributed to wrote the manuscript and data analysis. HG contributed to the methodology and material collection. Both authors contributed to the article and approved the submitted version.

## Conflict of Interest

The authors declare that the research was conducted in the absence of any commercial or financial relationships that could be construed as a potential conflict of interest.

## Publisher’s Note

All claims expressed in this article are solely those of the authors and do not necessarily represent those of their affiliated organizations, or those of the publisher, the editors and the reviewers. Any product that may be evaluated in this article, or claim that may be made by its manufacturer, is not guaranteed or endorsed by the publisher.
