# Corrigendum: New Breakthroughs and Innovation Modes in English Education in Post-pandemic Era

**DOI:** 10.3389/fpsyg.2022.899201

**Published:** 2022-04-12

**Authors:** Yumin Shen, Hongyu Guo

**Affiliations:** ^1^School of Foreign Languages, Zhejiang Gongshang University, Hangzhou, China; ^2^Graduate School of Education, University of Perpetual Help System DALTA, Metro Manila, Philippines

**Keywords:** post-pandemic, english teaching, dual-modality, facial expression, physiological signal

In the published article, there was an error in affiliations 1 and 2 as published. The order of the affiliations was incorrect. Instead of “^1^Graduate School of Education, University of Perpetual Help System DALTA, Las Piñas, Philippines” it should be “^1^School of Foreign Languages, Zhejiang Gongshang University, Hangzhou, China” and instead of “^2^School of Foreign Languages, Zhejiang Gongshang University, Hangzhou, China” it should be “^2^Graduate School of Education, University of Perpetual Help System DALTA, Metro Manila, Philippines.” The city “Las Piñas” has also been amended to be “Metro Manila”.

The authors apologize for this error and state that this does not change the scientific conclusions of the article in any way. The original article has been updated.

## Publisher's Note

All claims expressed in this article are solely those of the authors and do not necessarily represent those of their affiliated organizations, or those of the publisher, the editors and the reviewers. Any product that may be evaluated in this article, or claim that may be made by its manufacturer, is not guaranteed or endorsed by the publisher.

